# Menorrhagia at Puberty

**Published:** 1909-02-20

**Authors:** 


### Gynæcology.

### MENORRHAGIA AT PUBERTY.

It is by no means uncommon for some abnormality to attend the onset of menstruation and the first few years of menstrual life, just as excessive hæmorrhages may accompany the period preceding the menopause. It is, however, important to note that the hæmorrhages which accompany the onset of the menopause are much more likely to be, and in fact almost always are, the result of a pathological change in the uterus itself. This is not the case so often in cases of excessive hæmorrhage at the commencement of menstrual life. Of the abnormalities of menstruation at puberty, menorrhagia is the most serious for the patient, and the most difficult to cure. Such cases are by no means uncommon in hospital practice, though in the private practice of any individual they cannot be of more than occasional occurrence. As, however, there are several cases on record where such hæmorrhages have ended fatally, and many in which the patients have been reduced to an extreme condition of anæmia, it is important to recognise early and treat them appropriately.

The common history of such patients is that menstruation has started at about the usual time without any particular disturbance of the nervous system, but has been excessive from the commencement. The amount of loss is not always at its greatest at the first period, but may increase later; this is, in fact the common experience. In some cases, however, the first period may be a veritable flooding, making the patient anæmic, and then continuing as a constant, but less severe, loss for many weeks on end. These are the cases in which a fatal result has been recorded. In other patients, not only is the loss excessive, but also the interval between the periods is much shortened, the flow occurring perhaps every fourteen days, and continuing for a week or more. Many of the patients, in spite of the excessive losses, do not become seriously affected in general health, and may be quite well nourished, or even fat.

In the worst case of this kind seen by the writer the patient had always menstruated regularly, but in excessive quantities, until about the age of twenty. At this time she had a very serious hæmorrhage from the uterus occurring some days after a period had finished. This continued for several days, with constant loss and occasional "floodings," until her admission to hospital. She then seemed to be on the point of death, with a pulse between 160 and 180, sighing respiration, cold extremities, and great pallor. The uterus was plugged at once with sterilised gauze, and general restorative treatment adopted, with the result that the patient recovered. Previous drug treatment had had no effect whatever on the hæmorrhage. The age of this patient places her rather outside the usual limit of the cases under discussion, but nothing whatever could be found in the uterus itself to account for the hæmorrhage. There was not the slightest evidence of any recent abortion, and there was no growth. The patient admitted that she bled very easily from small wounds, and had suffered from epistaxis, so that there is reason to believe that some primary blood condition or abnormality of the vessels was to blame for the bleeding.

In discussing the causation of these conditions it is important to note first the absence of any gross lesion of the uterus. The occasional occurrence of a mucous polypus in a young girl or of a malignant growth of the cervix, grape-like sarcoma, has been the cause of bleeding in so small a proportion of the cases as to be almost negligible. At the same time it is important to make an examination to exclude growths of any kind. In the absence of a gross lesion the causes must be grouped under the headings—nervous system, blood and blood-vessels, and uterine abnormalities apart from growths.

Whatever the stimulus which starts menstruation, it would be expected to act in some way through the nervous system. At present it is generally believed to be primarily a bio-chemical stimulus connected with the internal secretion of the ovary. Whether this acts directly upon the uterine vessels, or through the central nervous system, is not at present clear; but it is obvious that the stimulus may be too great, leading to menorrhagia; or too small, leading to irregular menstruation with periods of amenorrhœa. This must be looked upon as a physiological error, and may be termed "excessive ovarian activity" just as "deficient ovarian activity" has always been quoted in the text-books as a cause of amenorrhœa.

Under the heading of the vascular system the only marked condition one can demonstrate is deficient coagulative power of the blood. This is undoubtedly a marked feature in some patients, and especially in those who suffer from chilblains and epistaxis. Even in these, however, it does not follow that they will also have menorrhagia, for the amount of loss does not depend solely on the blood itself, but also on the menstrual stimulus and the character of the uterine muscle. It has been suggested that the uterine capillaries are at fault, but there are no histological data to warrant this assumption. If the uterine muscle is badly developed, as is frequently the case, efficient control of the blood-vessels will be absent; unusual uterine congestion will then occur, and excessive hæmorrhage may result. Reasoned out in this way, the natural treatment of these patients will follow upon the theory of causation, and each case must be dealt with on its merits. For the excessive stimulus bromides and cannabis indica have been found to be useful, always remembering that constipation alone may help to cause general pelvic congestion. The administration of calcium lactate in gr. xxx. doses once a day for three days at fortnightly intervals (or, according to some observers, in smaller doses for longer periods), is the most efficient means we have of improving the coagulation time of the blood. Finally, the administration of ergot in ʒj doses of the liquid extract three times a day will cure those cases in which the uterine muscle requires stimulation.

